# The WISP1/Src/MIF Axis Promotes the Malignant Phenotype of Non-Invasive MCF7 Breast Cancer Cells

**DOI:** 10.3390/cells15020160

**Published:** 2026-01-15

**Authors:** Maria-Elpida Christopoulou, Panagiota Karamitsou, Alexios Aletras, Spyros S. Skandalis

**Affiliations:** 1 Biochemistry, Biochemical Analysis & Matrix Pathobiology Research Group, Laboratory of Biochemistry, Department of Chemistry, University of Patras, 26504 Patras, Greece; 2 Department of Pneumology, Medical Center-University of Freiburg, Faculty of Medicine, University of Freiburg, 79106 Freiburg, Germany

**Keywords:** breast cancer, WISP1, MIF, MMPs, hyaluronan, CD44, Src kinases

## Abstract

**Highlights:**

**What are the main findings?**

- WISP1 drives metastatic plasticity in ER+ breast cancer through Src-dependent induction of MIF.
- The WISP1/Src/MIF axis promotes EMT, extracellular matrix remodeling, and invasiveness of breast cancer cells.

**What are the implications of the main findings?**

- WISP1 regulates hyaluronan metabolism and proteolytic activity reshaping the tumor microenviroment.
- The WISP1/Src/MIF axis may serve as a therapeutic target in ER-positive breast cancer.

**Abstract:**

Breast cancer is a heterogeneous disease that exists in multiple subtypes, some of which still lack targeted and effective therapy. A major challenge is to unravel their underlying molecular mechanisms and bring to light novel therapeutic targets. In this study, we investigated the role of WNT-inducible signaling pathway protein 1 (WISP1) matricellular protein in the acquirement of an invasive phenotype by breast cancer cells. To this aim, we treated non-invasive MCF7 cells with WISP1 and assessed the expression levels of macrophage migration inhibitory factor (MIF) and its cellular receptor CD74. Next, we examined the expression of epithelial-to-mesenchymal transition (EMT) markers as well as molecular effectors of the tumor microenvironment, such as CD44, the main hyaluronan receptor that also acts as a co-receptor for MIF, the hyaluronan oncogenic network, and specific matrix metalloproteinases (MMPs) and their endogenous inhibitors, tissue inhibitors of metalloproteinases (TIMPs). The results showed that WISP1 potently induces the expression of MIF cytokine and affects the expression of specific extracellular matrix molecules with established roles in the promotion of malignant properties. Notably, Src kinases and MIF are critically involved in these processes. Collectively, the present study demonstrates for first time a WISP1/Src/MIF axis as well as its ability to induce an invasive phenotype in MCF7 cells and highlights novel cellular and molecular processes involved in the epithelial-to-mesenchymal transition and the development of invasive breast cancer. This suggests that specific cues from the tumor microenvironment can activate a migratory/invasive phenotype in a subpopulation of cells residing within the heterogeneous breast tumor.

## 1. Introduction

Breast cancer is characterized by significant heterogeneity, both between patients and within individual tumors, which poses substantial challenges for effective treatment strategies [1,2,3]. Breast tumors are categorized into various molecular subtypes based on hormone receptor status, HER2 expression, and proliferation markers; however, these classifications often fail to capture the full complexity of the disease. Within a single tumor, multiple molecular subtypes can coexist, further complicating treatment approaches [4]. This complexity is exacerbated by subtype plasticity, a phenomenon reflected by certain breast cancer cell lines, such as MCF7, which can switch between molecular profiles—such as from luminal-A to basal-like—depending on their microenvironment [5,6]. The tumor microenvironment (TME) plays a critical role in driving this plasticity. Components of the TME, including signaling molecules, extracellular matrix (ECM) proteins/carbohydrates, and immune cells, significantly influence tumor behavior and subtype expression [7,8,9]. As a result, traditional treatments targeting specific molecular subtypes often prove inadequate, particularly in cases where tumors exhibit the capacity for adaptation and evolution [10,11]. This highlights the urgent need for novel molecular targets and more sophisticated, combinatorial therapeutic strategies that account for the dynamic nature of tumor heterogeneity.

WNT-inducible signaling protein 1 (WISP1), also known as CCN4, is a matricellular protein in the CCN (Cysteine-rich, Connective Tissue Growth Factor, Nephroblastoma Overexpressed) family that modulates cell behavior through interactions with growth factors, receptors, and components of the extracellular matrix (ECM) [12]. Although WISP1 does not serve a structural role within the ECM, it functions as a regulatory mediator during embryonic development, wound repair, and tissue remodeling. In these physiological contexts, WISP1 contributes to tissue homeostasis by coordinating controlled cell proliferation, migration, differentiation, and ECM turnover [13,14,15]. When aberrantly upregulated, however, this homeostatic regulatory function becomes pathogenic: WISP1 overexpression disrupts ECM dynamics, enhances pro-tumorigenic signaling, and promotes epithelial-to-mesenchymal transition (EMT) [16,17]. In breast cancer specifically, WISP1 is overexpressed in primary tumors and correlates with tumor stage, size, and lymph node metastasis [18,19]. Functionally, WISP1 activates signaling pathways that drive EMT, tumor growth, and invasion, as reflected in changes in E-cadherin, N-cadherin, Snail, and β-catenin expression [20,21,22]. Additionally, WISP1 represses the tumor suppressor NDRG1, skews immune responses toward a type-2 phenotype, and enhances migration and invasion, particularly following PTEN loss [17,18]. Despite substantial evidence implicating WISP1 in breast cancer aggressiveness, the mechanisms by which WISP1 interfaces with other tumor microenvironment–derived factors remain insufficiently defined.

Another key player in the TME is Macrophage Migration Inhibitory Factor (MIF), a 12.5 kDa trimeric protein encoded by the *MIF* gene on human chromosome 22 [23]. Initially identified for its ability to inhibit macrophage migration, MIF is expressed by a wide range of immune and non-immune cells [24]. Elevated levels of MIF are found in several cancers, including breast cancer, where increased MIF concentrations in both tumor tissues and the bloodstream are associated with poor prognosis [25]. MIF is secreted into the extracellular space in response to inflammatory stimuli or stress, where it influences tumor progression via autocrine and paracrine signaling mechanisms. MIF primarily exerts its effects through interaction with the CD74 receptor, which is frequently upregulated in breast cancer and has been particularly associated with aggressive tumor features, including increased lymph-node invasion [26]. This association is more pronounced in more aggressive subtypes of breast cancer, such as triple-negative breast cancer (TNBC) [25]. CD74-mediated signaling is often co-activated by CD44, a major ECM receptor that also serves as a main cancer stem cell marker, leading to the activation of key oncogenic pathways, including the MAPK/ERK and PI3K/Akt pathways, which promote tumor cell proliferation, migration, and survival [26]. Additionally, MIF inhibits p53-dependent apoptosis, and promotes the secretion of factors such as vascular endothelial growth factor (VEGF) [27,28], facilitating angiogenesis and supporting the growth of metastatic lesions [29,30]. The interaction of MIF with the tumor stroma further contributes to an environment that favors tumor progression and metastasis [31]. Interestingly, while extracellular MIF correlates with poor outcomes, cytosolic MIF is linked to improved survival, reflecting a context-dependent function [32].

Breast cancer heterogeneity and therapy resistance are driven in large part by the complex interactions between tumor cells and their surrounding microenvironment, highlighting the need to identify molecular pathways that can be effectively targeted to modulate tumor behavior. Elevated levels of WISP1 and MIF correlate with aggressive disease features, reduced patient survival, and poor responses to conventional therapies, highlighting their potential not only as biomarkers for risk stratification but also as potential targets for therapeutic modulation. Given the emerging interplay between WISP1 and MIF [33] and their critical roles in shaping the TME to exacerbate tumor aggressiveness, this study aims to elucidate how this WISP1/MIF axis influences breast tumor plasticity and aggressiveness. To address this knowledge gap, the present study identifies and characterizes a previously unrecognized WISP1/Src/MIF signaling axis that regulates hyaluronan (HA) metabolism, EMT, and invasive behavior in ER^+^ breast cancer cells, providing novel mechanistic insights and potential avenues for therapeutic intervention. Importantly, both proteins engage signaling cascades that can be targeted pharmacologically, raising the possibility that disrupting this axis could attenuate phenotypic switching, suppress invasion, enhance patient responses to existing therapies, and provide a rationale for novel therapeutic strategies in breast cancer patients.

## 2. Materials and Methods

### 2.1. Cell Culture and Reagents

MCF7 (non-invasive, ER^+^) breast cancer cell line was obtained from the American Type Culture Collection (ATCC). MCF7 cells were routinely cultured in complete medium [Dulbecco’s Modified Eagle’s Medium supplemented with 10% fetal bovine serum and a cocktail of antimicrobial agents (100 IU/mL penicillin, 100 μg/mL streptomycin)] at 37 °C, 95% humidified air/5% CO_2_. Every two days, the medium was replaced with a fresh one. When approximately 80% cell confluency was reached, cells were trypsinized for 3 min with trypsin-EDTA 1× in PBS and seeded in new Petri dishes. All experiments were conducted in serum-free conditions unless otherwise stated.

Recombinant human WISP1 (endotoxin-free) and active MIF were used for cell culture treatments at the concentrations described in the Section 3. PP2 (Src kinase inhibitor) and ISO-1 (MIF inhibitor) were applied as indicated in each assay. All additional reagents used in the study were of analytical grade, and all materials were handled and stored according to standard laboratory procedures. Detailed information for all reagents used in this study, including recombinant proteins, inhibitors, primers, antibodies, and kits, is provided in Supplementary Table S1.

### 2.2. Determination of Secreted Hyaluronan Concentration

MCF7 cells were cultured under serum-free conditions as described above. At the desired time points, culture supernatants were collected, centrifuged at 1000× *g* for 5 min to remove cell debris, and stored at −80 °C until analysis. Secreted HA levels were quantified using the Hyaluronan ELISA Kit according to the manufacturer’s instructions. Absorbance was measured at 450 nm using a microplate reader (Infinite M200, Tecan, Männerdord, Switzerland), and HA concentrations were calculated based on a standard curve generated with known concentrations of HA.

### 2.3. Enzyme-Linked Immunosorbent Assays (ELISA)

Secreted levels of human MIF, MMP-1, MMP-2, MMP-9, MT1-MMP, TIMP-1, and TIMP-2 were quantified by ELISA using commercially available kits according to the manufacturers’ instructions (Supplementary Table S1). Standards and samples were prepared with kit-supplied diluents and diluted as needed to fall within each assay’s linear range. Absorbance was read at 450 nm with 540–570 nm reference correction using a microplate reader (Infinite M200, Tecan, Männerdord, Switzerland), and concentrations were calculated from 4-parameter logistic standard curves after background subtraction. Results are reported as pg/mL or ng/mL of conditioned medium and, where indicated, normalized to producing cell number or total cellular protein from matched wells. All data represent three independent experiments, with measurements performed in duplicate within each experiment. Statistical tests and replicate numbers are provided in the figure legends and in the Statistics subsection.

### 2.4. Detection of Phosphorylated Src Family Kinases by Capture ELISA

MCF7 cells were treated with WISP1 (500 ng/mL) for 24 h, washed with PBS, and lysed in ice-cold lysis buffer (700 μL per 90 mm dish). Lysates were clarified by centrifugation at 14,000× *g* for 30 min at 4 °C, and the supernatants were collected. Ninety-six-well polystyrene plates were coated overnight at 4 °C with antibodies specific for c-Src, Lyn, or Fyn (2 μg/mL in PBS; 100 μL/well). Plates were washed three times with PBS-T (PBS containing 0.05% Tween-20) and blocked with 1% BSA in PBS-T (200 μL/well) for 1 h at 37 °C. After three additional washes, cell lysates (100 μL/well; measured in duplicate) were added and incubated overnight at 4 °C. Plates were then washed five times with PBS-T, and bound phosphorylated proteins were detected using an HRP-conjugated anti-phosphotyrosine antibody (1:2000 in PBS-T containing 0.25 M NaCl; 100 μL/well) for 2 h at 37 °C. After five washes with PBS-T containing 0.25 M NaCl, TMB substrate (100 μL/well) was added, and color development was allowed for 15 min at room temperature in the dark. The reaction was stopped with 2 M H_2_SO_4_ (100 μL/well), and absorbance was measured at 450 nm using a microplate reader (Infinite M200, Tecan).

### 2.5. Immunofluorescence Microscopy

MCF-7 cells (3 × 10^4^) were seeded on sterile glass coverslips in complete medium and incubated for 24 h, followed by overnight serum starvation. Cells were then stimulated with recombinant WISP1 (500 ng/mL) in the presence or absence of PP2 or ISO-1, or with recombinant MIF (150 ng/mL), for 24 h in serum-free medium. Following stimulation, cells were fixed in 4% paraformaldehyde in PBS for 15 min at room temperature, permeabilized with 0.05% Triton X-100/PBS-Tween 0.01% for 10 min, and blocked with 3% bovine serum albumin (BSA)/PBS-Tween 0.01% for 1 h. Cells were incubated overnight at 4 °C with a primary antibody against E-cadherin diluted in 1% BSA/PBS-Tween 0.01%, followed by an Alexa Fluor 488-conjugated secondary antibody for 1 h at 37 °C in the dark. F-actin was visualized using Phalloidin-iFluor™ 488 Conjugate (1:40 dilution). Nuclei were counterstained and coverslips mounted with Vectashield Antifade Mounting Medium with DAPI. Between each step after fixation, cells were washed three times with PBS-Tween 0.01%. Images were acquired using a fluorescence phase-contrast microscope (Olympus CKX41, QImaging Micro Publisher 3.3RTV) at 40× magnification. All experiments were performed in three independent biological replicates, with measurements in duplicate.

### 2.6. Western Blotting

For analysis of secreted MIF, equal protein concentrations from conditioned medium were determined using the Bradford assay and enriched by ammonium sulfate precipitation [(NH_4_)_2_SO_4_, 50% saturation]. Precipitates were dissolved in Laemmli sample buffer containing β-mercaptoethanol, boiled for 5 min, and subjected to SDS–PAGE on 15% polyacrylamide gels. Proteins were transferred to PVDF membranes, blocked, and immunoblotted using an anti-MIF antibody. For analysis of cellular proteins, MCF-7 cells were lysed in RIPA buffer supplemented with protease and phosphatase inhibitor cocktail (1×), on ice for 30 min with intermittent vortexing. Lysates were centrifuged at 10,000 rpm for 10 min at 4 °C, and supernatants were collected. Protein concentrations were determined using a BCA Protein Assay Kit. Equal amounts of protein were separated by SDS–PAGE on 10% polyacrylamide gels and transferred to PVDF membranes. Membranes were blocked with 5% (*w*/*v*) non-fat dry milk in PBS containing 0.05% Tween-20 (PBS-T) for 1 h at room temperature, then incubated overnight at 4 °C with primary antibodies: E-cadherin, NDRG1, and α-tubulin. After three washes with PBS-T, membranes were incubated for 1 h at room temperature with HRP-conjugated secondary antibodies anti-rabbit IgG; or anti-mouse IgG, for E-cadherin and α-tubulin. NDRG1 was detected directly using HRP-conjugated streptavidin without additional secondary antibody. Immunoreactive protein bands were visualized using enhanced chemiluminescence (Immunobilon^®^ Crescendo Western HRP Substrate). Band intensity was quantified by densitometry using ImageJ software (version 1.54g; National Institutes of Health, Bethesda, MD, USA; https://imagej.net) and expressed in arbitrary units (pixels). All Western blots were performed in three independent experiments, with duplicate measurements where applicable.

### 2.7. Quantitative Real-Time PCR

Total RNA was extracted from MCF7 cells using a Nucleo Spin RNA kit according to the manufacturer’s instructions. cDNA synthesis was performed using the Prime Script RT Reagent Kit as per the provided protocol. Quantitative real-time PCR (qPCR) analysis was conducted using the KAPA SYBR FAST qPCR Master Mix (2×) kit, following the manufacturer’s guidelines. Assays were performed in three independent experiments, with each sample measured in duplicate. Reactions were run on a Rotor-Gene Q detection system (Qiagen, Hilden, Germany) in a total volume of 20 μL, comprising 10 μL KAPA SYBR FAST qPCR Master Mix (2×), 0.5 μL of each primer at a concentration of 8 μM, 1–2 μL of cDNA (0.5–20 ng), and 7 or 8 μL of dH_2_O. The cycling conditions included 3 min enzyme activation at 95 °C, followed by 40 cycles at 95 °C for 3 s and 50–60 °C for 20 s. GAPDH was used as an internal standard. Relative expression of different gene transcripts was calculated by the ΔΔCt method. The Ct of any gene of interest was normalized to the Ct of the normalizer (GAPDH). Fold changes (arbitrary units) were determined as 2^−ΔΔCt^. Genes of interest and utilized primers are presented in Table 1.

### 2.8. Wound Healing Assay

MCF7 cells were cultured in 6-well plates in complete medium until confluent monolayers. Cells were then serum starved for 16 h and the cell monolayer was scratched using a pipette tip. Cells were washed two times with PBS followed by the addition of serum free DMEM containing the desired factors [WISP1 (500 ng/mL), MIF (150 ng/mL), PP2 (1 μΜ), ISO-1 (100 μΜ)] plus cytarabine (10 μM). For the pre-treatment with PP2 and ISO-1 inhibitors prior to WISP1 treatment, cells were pre-incubated with the inhibitors in serum free medium for 30 min. The cells were then incubated and photographed at various time points (0 h, 6 h, 12 h, 24 h, 48 h) using a colour digital camera (CMOS) mounted on a phase contrast microscope (OLYMPUS CKX41, QImaging Micro Publisher 3.3RTV) through a 10× objective. The images were quantified by measuring the wound area with Image J 1.50b Launcher Symmetry Software (version 1.54g; National Institutes of Health, Bethesda, MD, USA; https://imagej.net). All wound healing experiments were performed in three independent biological replicates, with multiple fields per well measured as technical replicates. Wound closure values were normalized to the initial wound area at time 0 h and, when applicable, to the untreated control (0.1% FBS).

### 2.9. MTT Assay

The MTT assay was used to assess the effect of WISP1/MIF axis in the viability and proliferation of MCF7 cells. MCF7 cells were seeded in 96-well plates at a density of 1 × 10^4^ cells per well and allowed to adhere for 24 h. Cells were then treated with various concentrations of test compounds or inhibitors, diluted in serum-free DMEM. After 24 h of incubation, the medium was removed, and the wells were washed with PBS to remove any residual test substances. Next, 100 μL of MTT solution (0.5 mg/mL in PBS) was added to each well and incubated for 4 h at 37 °C. The formazan crystals formed in living cells were dissolved by adding 100 μL of DMSO to each well. The absorbance was measured at 570 nm using a microplate reader. The results were expressed as the percentage of viable cells compared to control cells treated with vehicle (DMSO) alone. The MTT assay reflects mitochondrial metabolic activity as an indicator of cell viability and may register changes in metabolic activity as altered viability, even in the absence of overt cell death or changes in cell number. All MTT assays were performed in three independent biological replicates, with each condition measured in triplicate. Results are expressed as percent viable cells relative to vehicle-treated controls.

### 2.10. Transwell Invasion Assay

Cell invasion assays were performed using 48-well Transwell inserts with 5 μm pores (Corning). Inserts were coated with type I collagen (2.4 mg/mL in PBS) and allowed to polymerize at 37 °C for 1 h. MCF7 cells were serum-starved overnight in medium containing 0.1% FBS. For stimulation experiments, adherent cells were treated with WISP1 (500 ng/mL), recombinant human MIF (150 ng/mL), or pre-treated for 1 h with PP2 (1 μM) or ISO-1 (100 μM) prior to WISP1 stimulation for 24 h in medium containing 0.1% FBS. Following treatment, cells were detached using 0.05% trypsin-EDTA (Gibco), neutralized, pelleted, and resuspended in serum-free medium containing the same treatment conditions prior to seeding. Cells were then seeded into the upper chamber of collagen-coated 48-well inserts at 4.0 × 10^4^ cells per insert. The lower chamber was filled with medium supplemented with 10% FBS, which served as the chemoattractant. The plates were incubated at 37 °C for 24 h and 48 h after seeding. At each time point, non-invaded cells on the upper membrane surface were removed, invading cells on the underside were fixed, stained (crystal violet), imaged, and quantified. Invasion was evaluated by counting cells in multiple randomly selected fields or by measuring stained area, and data were normalized to control wells.

### 2.11. Statistical Analysis

Each experiment was performed at least three times and measurements within each experiment were performed in duplicate. Data are presented as mean ± standard deviation (SD). Statistical differences were evaluated using two-way analysis of variance (ANOVA) followed by Tukey’s multiple comparisons test. Differences were considered statistically significant at *p* ≤ 0.05. All analyses and graphs were performed using GraphPad Prism 10.5.0 software.

## 3. Results

### 3.1. WISP1 Induces MIF Secretion in MCF7 Breast Cancer Cells Through Src Kinase-Dependent Mechanisms

**Figure 1:**
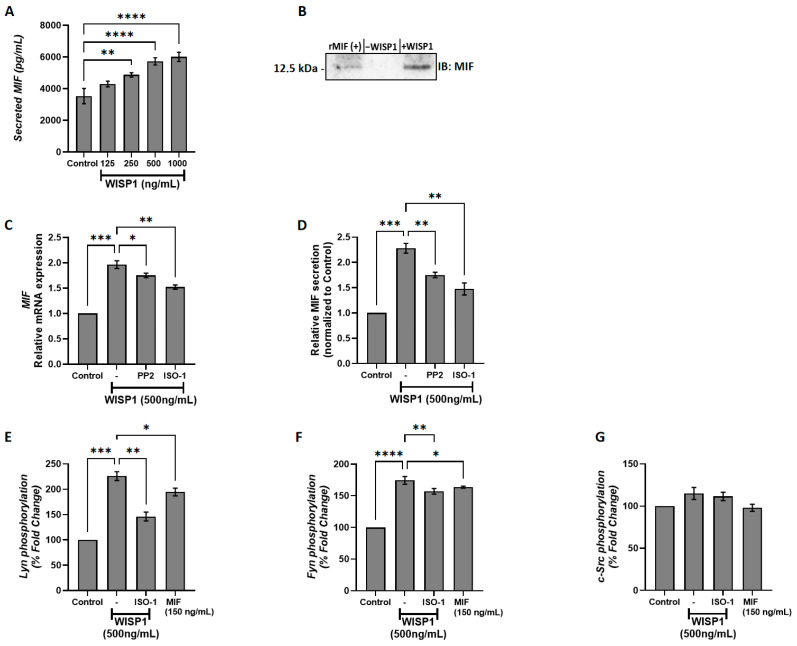
WISP1 induces MIF expression and secretion via Src kinase activation. (**A**) MCF7 cells were cultured with increasing concentrations of recombinant WISP1 (0, 125, 250, 500, and 1000 ng/mL) for 24 h, and secreted MIF protein levels were measured in cell culture supernatants by ELISA. (**B**) Western blot analysis of secreted MIF in supernatants from MCF7 cells. Cells were untreated (–WISP1) or treated with WISP1 (+WISP1). Recombinant MIF (rMIF) was used as a positive control. Cell culture supernatants were collected and immunoblotted with an anti-MIF antibody. A band at ~12.5 kDa indicates secreted MIF. (**C**,**D**) MCF7 cells were pretreated for 30 min with the Src kinase inhibitor PP2 (1 μM) or the MIF inhibitor ISO-1 (100 μM), followed by stimulation with WISP1 (500 ng/mL) for 24 h. (**C**) MIF mRNA expression was analyzed by quantitative PCR (qPCR). (**D**) Secreted MIF protein levels in the cell culture supernatants were quantified by ELISA. (**E**–**G**) In situ kinase assays of (**E**) Lyn, (**F**) Fyn, and (**G**) c-Src phosphorylation in cells treated with WISP1 (500 ng/mL) in the presence or absence of the MIF inhibitor ISO-1 (100 μM) or recombinant MIF (150 ng/mL). Data are mean ± SD from *n* = 3 independent experiments, each performed in duplicate. Statistical analysis was performed as described in Methods (one-way or two-way ANOVA with Tukey’s multiple comparisons test, as appropriate). * *p* < 0.05; ** *p* < 0.01; *** *p* < 0.001; **** *p* < 0.0001.

Our previous work demonstrated a functional link between WISP1 and MIF in normal lung fibroblasts [33]. To investigate whether WISP1 also regulates MIF expression in breast cancer, non-invasive ER^+^ MCF7 cells were treated with increasing concentrations of recombinant human WISP1 (125, 250, 500, and 1000 ng/mL) for 24 h, and secreted MIF levels were quantified by ELISA. WISP1 induced a dose-dependent increase in MIF secretion, with moderate rises at 125 ng/mL and 250 ng/mL, and a significant increase of ~2-fold at 500 ng/mL (*p* < 0.01) compared to untreated controls (Figure 1A). No further increase was detected at 1000 ng/mL. These results indicate that WISP1 positively regulates MIF secretion, suggesting that MIF may act as a downstream mediator of WISP1-induced cellular responses in breast cancer cells. To further validate these findings, we performed Western blotting on cell supernatants. Equal amounts of total protein were loaded, and recombinant MIF was included as a positive control, revealing a clear increase in secreted MIF that migrated as a single band at ~12.5 kDa (Figure 1B). To determine the underlying molecular mechanisms, MCF7 cells were pre-treated for 30 min with either the Src kinase inhibitor PP2 or the MIF inhibitor ISO-1 prior to WISP1 stimulation (500 ng/mL, 24 h). Both inhibitors significantly attenuated WISP1-induced MIF expression, with PP2 reducing MIF levels by ~20% at the mRNA level and ~23% at the protein level, while ISO-1 reduced mRNA and protein levels by ~23% and ~35%, respectively, compared to WISP1 treatment alone (Figure 1C,D), indicating that Src kinases and MIF itself are implicated in WISP1-mediated upregulation of MIF in breast cancer cells. Finally, to further confirm the involvement of Src kinases, we assessed the phosphorylation status of key Src family members (c-Src, Lyn, and Fyn) by capture ELISA using phospho-tyrosine–specific detection. WISP1 treatment increased Src family phosphorylation, whereas PP2 pre-treatment effectively blocked this effect, demonstrating that WISP1 activates Src kinases upstream and that Src activity is required for WISP1-induced MIF upregulation. WISP1 stimulation significantly induced the phosphorylation of Lyn (mainly) (Figure 1E) and Fyn kinases (Figure 1F), but not c-Src (Figure 1G), with Lyn phosphorylation increasing approximately 2-fold and Fyn phosphorylation increasing approximately 1.5- to 2-fold relative to controls, indicating these kinases as upstream mediators of WISP1-induced secretion of MIF in MCF7 cells. To determine whether this phosphorylation depends on MIF activity, cells were pre-treated with ISO-1, which reduced Lyn phosphorylation by ~35% and Fyn phosphorylation by ~20% compared to WISP1 alone. Notably, the inhibitory effect of ISO-1 suggests that MIF expression is sustained, at least in part, via a self-reinforcing, ISO-1-inhibitable feedback loop. This finding supports the notion that MIF not only acts downstream of WISP1 but may also contribute to the maintenance of its own expression. These findings are consistent with our previous studies in lung fibroblasts [33], suggesting a conserved regulatory mechanism for WISP1-induced MIF expression across different cell types.

### 3.2. Implication of Src Kinases and MIF in WISP1-Mediated Changes in CD44/CD74 Receptors Expression

**Figure 2:**
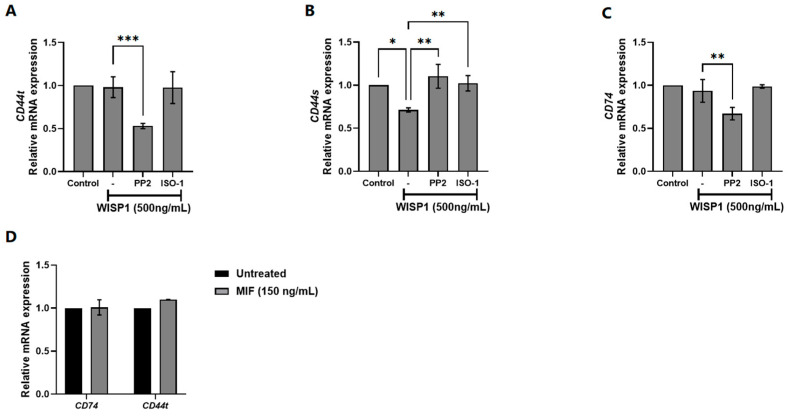
Effect of WISP1 on CD74 and CD44 mRNA levels. MCF7 cells were treated for 24 h with WISP1 (500 ng/mL). Where indicated, cells were pre-treated for 1 h with the Src kinase inhibitor PP2 (1 μM) or the MIF inhibitor ISO-1 (100 μM) prior to WISP1 stimulation; a recombinant human MIF (rhMIF, 150 ng/mL) condition was included as a comparator. (**A**–**C**) Relative mRNA expression of CD74 (**A**), total CD44 (**B**), and CD44s (**C**) under WISP1 ± inhibitor conditions, quantified by qPCR, normalized to GAPDH, and expressed relative to untreated control. (**D**) Relative mRNA expression of CD74 and total CD44 in cells treated with rhMIF (150 ng/mL) for 24 h, analyzed as above. Data are mean ± SD from *n* = 3 independent experiments, each performed in duplicate. Statistical analysis was performed as described in Methods (one-way or two-way ANOVA with Tukey’s multiple comparisons test, as appropriate). * *p* < 0.05; ** *p* < 0.01; *** *p* < 0.001.

Given the ability of WISP1 to induce MIF expression in MCF7 cells, we next examined whether WISP1 also modulates the expression of MIF cellular receptors, specifically CD44 and CD74. MCF7 cells were treated with WISP1 (500 ng/mL) in the presence or absence of the Src kinase inhibitor PP2 or the MIF inhibitor ISO-1. qPCR analysis revealed that WISP1 had no significant effect on the mRNA levels of total CD44 (CD44t: combined CD44s/CD44v) (Figure 2A) but significantly downregulated the CD44s isoform (Figure 2B). CD74 expression remained unchanged upon WISP1 stimulation (Figure 2C). Pre-treatment with PP2 prior to WISP1 exposure led to marked reduction in CD44t (Figure 2A) and CD74 (Figure 2C) expression compared to untreated or WISP1-treated cells, while reversed the WISP1-mediated suppression of CD44s (Figure 2B). Importantly, treatment with PP2 alone did not significantly affect CD74 mRNA levels compared to untreated controls (Supplementary Figure S1). In contrast, ISO-1 pre-treatment followed by WISP1 stimulation did not alter the expression of CD44t, CD44s, or CD74 (Figure 2A–C), indicating that MIF induction by WISP1 is not implicated in the transcriptional regulation of its receptors. Consistently, treatment with recombinant MIF (150 ng/mL) failed to induce changes in CD44t or CD74 mRNA expression (Figure 2D). Overall, our data indicate that while WISP1 activates MIF expression, this induction does not drive changes in the transcription of its major receptors CD44 and CD74. Neither pharmacological inhibition of MIF nor stimulation with recombinant MIF altered receptor mRNA levels, arguing against an autocrine feedback loop in which MIF enhances its own signaling by increasing receptor availability. These findings suggest that WISP1-induced MIF may signal through alternative mechanisms, such as modifying receptor activity rather than expression, or engaging additional downstream pathways independent of CD44/CD74 transcription.

### 3.3. WISP1 Promotes EMT Features in Breast Cancer Cells Through Src and MIF Activities

**Figure 3:**
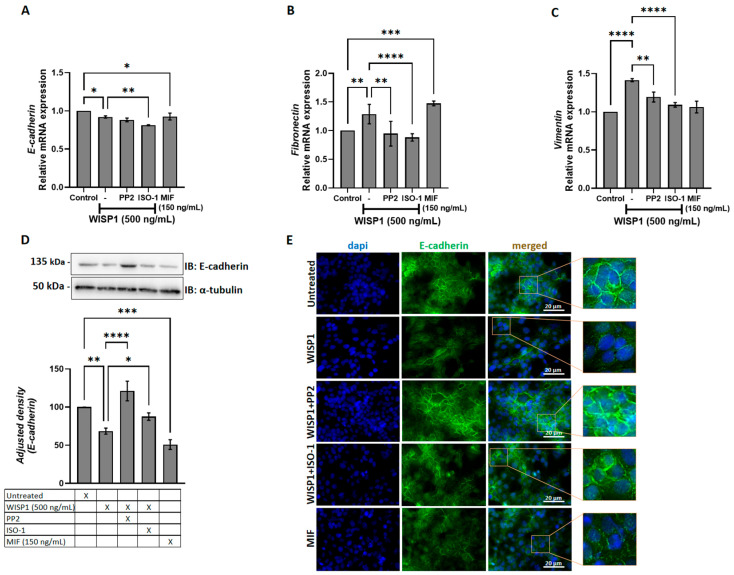
WISP1 and MIF modulate EMT marker expression via Src kinases. MCF7 cells were treated for 24 h with WISP1 (500 ng/mL). Where indicated, cells were co-treated with the Src kinase inhibitor PP2 (1 μM), the MIF inhibitor ISO-1 (100 μM), or recombinant human MIF (100 ng/mL). (**A**–**C**) Relative mRNA expression of E-cadherin (**A**), fibronectin (**B**), and vimentin (**C**) quantified by qPCR, normalized to GAPDH, and expressed relative to untreated control. (**D**) Representative Western blot of E-cadherin in cell lysates from the same treatment conditions; α-tubulin served as a loading control. Densitometric quantification of band intensities is shown below the blots. (**E**) Representative immunofluorescence images of E-cadherin (green) and nuclei (DAPI, blue) under the indicated treatments; insets show higher-magnification views highlighting cell–cell junctions. Scale bars, 20 μm. Data are mean ± SD from *n* = 3 independent experiments, each performed in duplicate. Statistical analysis was performed as described in Methods (one-way or two-way ANOVA with Tukey’s multiple comparisons test, as appropriate). * *p* < 0.05; ** *p* < 0.01; *** *p* < 0.001; **** *p* < 0.0001.

WISP1 has been implicated in the induction of EMT in several cancers, including oral squamous cell carcinoma, prostate, and breast cancer [21,34,35]. To assess whether WISP1 induces similar effects in non-invasive MCF7 cells, we treated cells with recombinant WISP1 (500 ng/mL) for 24 h. The results showed that WISP1 significantly decreased E-cadherin mRNA expression levels (Figure 3A). To validate the mRNA changes, E-cadherin protein expression was assessed by Western blotting of whole-cell lysates. Western blot analysis revealed a decrease in E-cadherin protein expression following WISP1 treatment, with densitometric quantification showing a 30% decrease compared to control. Protein levels were normalized to α-tubulin, which served as loading control (Figure 3D). This decrease was accompanied by disrupted E-cadherin junctional localization as shown by immunofluorescence (Figure 3E). On the other hand, WISP1 treatment resulted in significant upregulation of the mesenchymal markers fibronectin and vimentin at the mRNA level (Figure 3B,C), reflecting the acquisition of mesenchymal traits. The induction of these mesenchymal markers indicates that WISP1 not only suppresses epithelial identity but also actively drives mesenchymal programming, reinforcing its role as a potent EMT regulator. The marked increase in fibronectin and vimentin further supports a shift toward a more motile and invasive phenotype. Treatment with recombinant human MIF (100 ng/mL) resulted in similar effects, whereas inhibition of Src with PP2 (10 μM) or blockade of MIF activity with ISO-1 (50 μM) partially restored E-cadherin expression, junctional localization, and epithelial morphology (Figure 3E), while reversing the WISP1-induced upregulation of fibronectin and vimentin (Figure 3B,C). These results indicate that WISP1 initiates a partial EMT in MCF7 cells, characterized by the acquisition of mesenchymal features without complete loss of epithelial identity. Importantly, these WISP1-driven phenotypic changes rely heavily on Src kinase and MIF signaling. Inhibition of either pathway rescues epithelial and mesenchymal marker expression and partially restores epithelial junctional organization, demonstrating that Src and MIF are essential for the full execution and maintenance of the WISP1-induced EMT state.

### 3.4. WISP1/MIF Axis Affects the Oncogenic Hyaluronan Network

**Figure 4:**
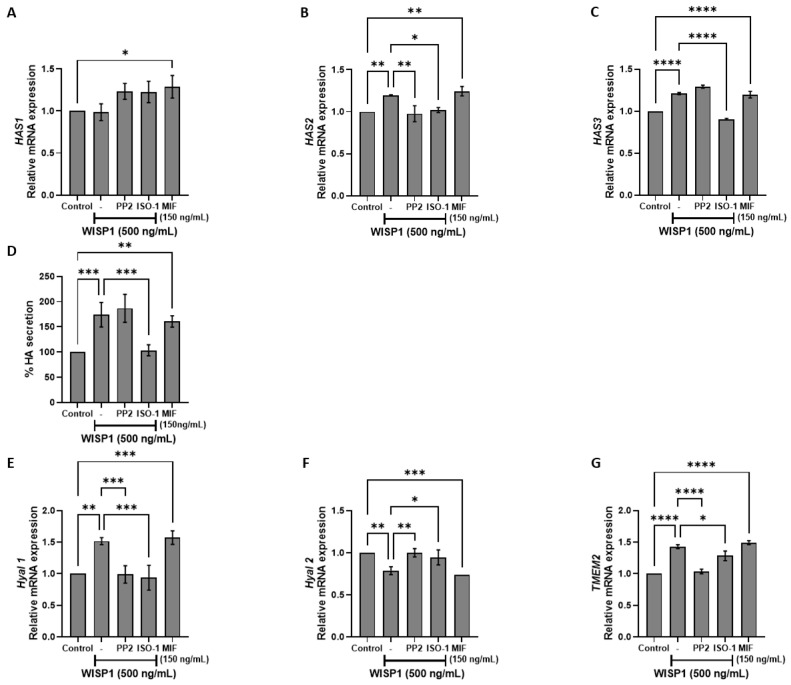
WISP1 regulates hyaluronan metabolism via Src and MIF signaling. MCF7 cells were treated with WISP1 (500 ng/mL) in the presence or absence of the Src family kinase inhibitor PP2 (1 μM), the MIF inhibitor ISO-1 (100 μM), or recombinant human MIF (150 ng/mL). (**A**–**C**,**E**–**G**) Gene expression analysis of HAS1 (**A**), HAS2 (**B**), HAS3 (**C**), HYAL1 (**E**), HYAL2 (**F**), and TMEM2 (**G**) by qPCR, normalized to GAPDH and expressed relative to untreated control. (**D**) Secreted hyaluronic acid (HA) quantified by ELISA from the same treatment conditions; HA is expressed as percent of untreated control and normalized to cell number. Data are mean ± SD from *n* = 3 independent experiments, each performed in duplicate. Statistical analysis was performed as described in Methods (one-way or two-way ANOVA with Tukey’s multiple comparisons test, as appropriate). * *p* < 0.05; ** *p* < 0.01; *** *p* < 0.001; **** *p* < 0.0001.

Dysregulation of HA metabolizing enzymes, including hyaluronan synthases (HASes) and hyaluronidases (HYALs), contributes to poor prognosis and resistance to chemotherapy in several cancers [36]. To examine the implication of WISP1 in extracellular matrix (in particular hyaluronan network) remodeling during tumor progression, MCF7 cells were treated with WISP1 in the absence or presence of Src kinase and MIF inhibitors. The results revealed that WISP1 upregulated HAS2 and HAS3, with no effect on HAS1 (Figure 4A–C). Pre-treatment with ISO-1 abolished WISP1-induced HAS2 and HAS3 expression, whereas HAS2 was also suppressed by PP2 (Figure 4A–C), indicating that WISP1 promotes HA synthesis through MIF (mainly) and Src signaling. In agreement with this, treatment with recombinant human MIF resulted in the upregulation of all HASes (Figure 4A–C). Consistent with the HAS transcriptional changes, WISP1 markedly increased HA secretion by ~1.8-fold compared to control cells (Figure 4D). Notably, MIF alone was also sufficient to elevate HA levels by ~1.6-fold. Pre-treatment with the MIF inhibitor ISO-1 blocked this increase, reducing HA secretion by ~40%, whereas Src inhibition with PP2 had no effect (Figure 4D). These results demonstrate that WISP1 upregulates HA secretion through a MIF-dependent mechanism. To assess the impact of WISP1 on hyaluronan turnover, we examined the expression of key hyaluronidases involved in HA degradation, namely Hyal1, Hyal2, and transmembrane protein 2 (TMEM2). WISP1 treatment significantly increased the mRNA levels of Hyal1 and TMEM2 (Figure 4E,G), while both WISP1 and MIF downregulated Hyal2 expression (Figure 4F). The WISP1-mediated induction of Hyal1 and TMEM2 was blocked by ISO-1 and PP2 inhibitors, while the reverse effect was observed for Hyal2 (Figure 4E–G) suggesting that WISP1 regulates HA turnover through the actions of Src kinases and MIF.

### 3.5. WISP1/MIF Axis Modulates MMPs/TIMPs and ECM Remodeling

**Figure 5:**
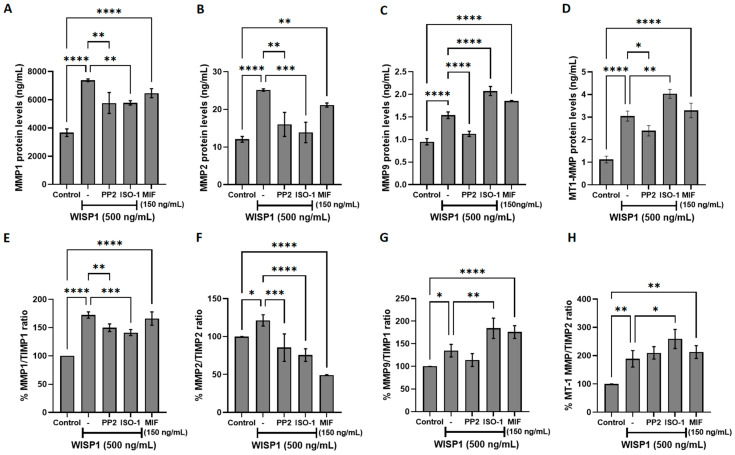
Effects of WISP1 and MIF on MMP protein levels and MMP/TIMP ratios. MCF7 cells were treated for 24 h with WISP1 (500 ng/mL). Where indicated, cells were co-treated with the Src family kinase inhibitor PP2 (1 μM) or the MIF inhibitor ISO-1 (100 μM); a recombinant human MIF (rhMIF, 150 ng/mL) condition was included as a comparator. (**A**–**D**) ELISA quantification of MMP1 (**A**), MMP2 (**B**), MMP9 (**C**), and MT1-MMP (**D**) protein levels (ng/mL). (**E**–**H**) Ratios of MMP1/TIMP1 (**E**), MMP2/TIMP2 (**F**), MMP9/TIMP1 (**G**), and MT1-MMP/TIMP2 (**H**), calculated from ELISA values and expressed as percentage relative to untreated control (set to 100%). Data are mean ± SD from *n* = 3 independent experiments, each performed in duplicate. Statistical analysis was performed as described in Methods (one-way or two-way ANOVA with Tukey’s multiple comparisons test, as appropriate). * *p* < 0.05; ** *p* < 0.01; *** *p* < 0.001; **** *p* < 0.0001.

To further investigate the role of WISP1 in extracellular matrix remodeling, we examined its effects on matrix metalloproteinases (MMPs) and their endogenous tissue inhibitors (TIMPs), which are key regulators of extracellular matrix dynamics and tumor invasion [37,38]. Non-invasive MCF7 cells were treated with WISP1 for 24 h, and ELISA assays were performed to quantify MMP protein levels. WISP1 significantly increased the secretion of MMP1, MMP2, MMP9, and MT1-MMP (Figure 5A–D), with MMP1 and MMP2 increasing approximately 2-fold, MMP9 by ~1.7-fold, and MT1-MMP by ~3-fold relative to untreated controls. Supporting qPCR data are provided in Supplementary Figure S1, but given the divergence between mRNA and protein regulation, we focused our analysis on protein level outcomes, which more directly reflect functional MMPs in the extracellular matrix. Pharmacological inhibition of Src with PP2 or MIF with ISO-1 attenuated WISP1-induced MMP1 and MMP2 secretion, while their effects on MT1-MMP were modest. Furthermore, treatment with recombinant human MIF resulted in the upregulation of all examined MMPs (Figure 5A–D). However, WISP1-induced MMP9 secretion was suppressed by Src inhibition but increased following MIF activity inhibition (Figure 5C). Specifically, PP2 reduced WISP1-induced MMP1 and MMP2 levels by approximately 22% and 36%, respectively, while ISO-1 reduced them by ~24% and ~45%. For MMP9 and MT1-MMP, PP2 decreased levels by ~27% and ~21%, whereas ISO-1 increased them by ~35% and ~37%, respectively. Furthermore, treatment with recombinant human MIF resulted in the upregulation of all examined MMPs (Figure 5A–D), ranging from approximately 1.7-fold to 2.6-fold depending on the MMP. Consistent with these findings, WISP1 treatment significantly increased MMP1/TIMP1 (~1.7-fold), MMP2/TIMP2 (~1.2-fold), MMP9/TIMP1 (~1.3-fold), and MT1-MMP/TIMP2 (~1.9-fold) ratios, suggesting a shift toward proteolytic activity (Figure 5E–G). Src kinase inhibition with PP2 reduced these WISP1-induced ratios by approximately 23% for MMP1/TIMP1, 30% for MMP2/TIMP2, and a smaller reduction of ~15% for MMP9/TIMP1 (Figure 5E–G). In contrast, MIF inhibition with ISO-1 selectively decreased MMP1/TIMP1 by ~17% and MMP2/TIMP2 by ~38%, but increased MMP9/TIMP1 by ~37% and MT1-MMP/TIMP2 by ~47% relative to WISP1 treatment alone (Figure 5E–G). Supporting qPCR data regarding TIMPs mRNA expression are provided in Supplementary Figure S2.

### 3.6. WISP1 Promotes Breast Cancer Cell Viability via Src Kinases and MIF

**Figure 6:**
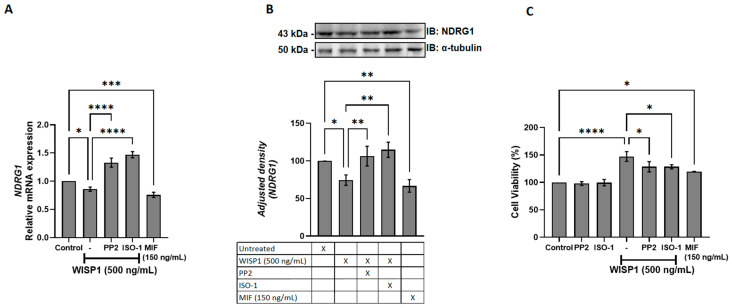
WISP1 regulates NDRG1 expression and cancer cell viability via Src kinases and MIF. MCF7 cells were treated for 24 h with WISP1 (500 ng/mL) in the presence or absence of the Src kinase inhibitor PP2 (1 μM), the MIF inhibitor ISO-1 (100 μM), or recombinant human MIF (100 ng/mL). (**A**) NDRG1 mRNA levels quantified by qPCR, normalized to GAPDH, and expressed relative to untreated control. (**B**) Western blot analysis of NDRG1 protein; α-tubulin served as a loading control. Densitometric quantification of band intensities is shown below the blots. (**C**) Cell viability measured by MTT assay at 24 h. Data are mean ± SD from *n* = 3 independent experiments, each performed in duplicate. Statistical analysis was performed as described in Methods (one-way or two-way ANOVA with Tukey’s multiple comparisons test, as appropriate). * *p* < 0.05; ** *p* < 0.01; *** *p* < 0.001; **** *p* < 0.0001.

To determine whether WISP1 and MIF regulate NDRG1 expression and impact tumor cell survival, MCF7 cells were treated with recombinant WISP1, with or without Src kinase (PP2) or MIF (ISO-1) inhibitors, alongside parallel stimulation with recombinant MIF. qPCR and Western blot analysis revealed a clear reduction in NDRG1 protein following WISP1 stimulation. Specifically, treatment with recombinant WISP1 (500 ng/mL) produced a marked decrease in the intensity of the NDRG1 band, with densitometric quantification showing a ~45–60% reduction compared to untreated controls (Figure 6A,B). This reduction did not affect α-tubulin, which served as loading control. Co-treatment with the Src inhibitor PP2 or the MIF antagonist ISO-1 reversed the WISP1-mediated reduction of NDRG1, restoring protein levels to approximately 85–95% of control intensity. This restoration was clearly visible on the blot as enhanced NDRG1 band intensity in the inhibitor groups.

In parallel, recombinant MIF (150 ng/mL) alone decreased NDRG1 protein levels by approximately 30–40%, resulting in a visibly weaker band compared to untreated cells, consistent with mRNA suppression. WISP1 enhanced cell viability relative to controls (Figure 6C), an effect partially reversed by Src or MIF inhibition. Accordingly, recombinant MIF increased cell viability (Figure 6C). These results establish WISP1 and MIF as negative regulators of NDRG1 and demonstrate that Src and MIF signaling are required for the observed WISP1-mediated effects on breast cancer cell survival.

### 3.7. WISP1 Promotes Breast Cancer Cell Migration Through Src Kinases and MIF

**Figure 7:**
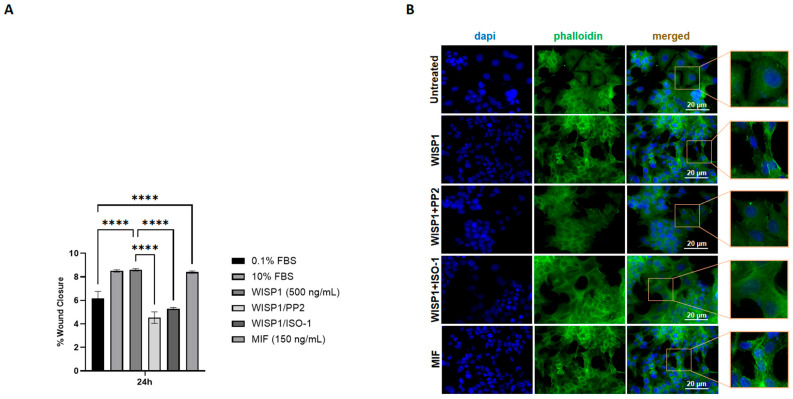
Effects of WISP1 and MIF on cancer cell motility. MCF7 cells were treated with WISP1 (500 ng/mL), MIF (150 ng/mL), or pre-treated with the Src kinase inhibitor PP2 (1 μM) or the MIF inhibitor ISO-1 (100 μM) prior to WISP1 stimulation. The 0.1% FBS condition represents the untreated control, while a high-serum positive control (10% FBS) was included. (**A**) Migration was assessed by wound-healing assay over 48 h, with data specifically shown for the 24 h time point; wound closure is expressed as the percentage of the initial wound area at 0 h. (**B**) Representative immunofluorescence images of F-actin (phalloidin, green) and nuclei (DAPI, blue) under the indicated treatments; insets show higher-magnification views highlighting cytoskeletal organization. Scale bars, 20 μm. Data are mean ± SD from *n* = 3 independent experiments, each performed in duplicate. Statistical analysis was performed as described in Methods (one-way or two-way ANOVA with Tukey’s multiple comparisons test, as appropriate). **** *p* < 0.0001.

Wound healing assays were performed over a 0–48 h time course with full quantitative analysis and representative microscopic images provided in Supplementary Figures S3 and S4, respectively. At 24 h, these assays demonstrated that WISP1 (500 ng/mL) significantly induced migration of MCF7 cells, resulting in an approximately 2-fold increase in wound closure compared to control cells (Figure 7A). Similarly, recombinant MIF (150 ng/mL) increased migration, with an approximately 2-fold increase (Figure 7A). The presence of the Src kinase inhibitor PP2 significantly attenuated WISP1-mediated migration at 24 h, reducing wound closure by approximately 47% (Figure 7A). Likewise, the MIF inhibitor ISO-1 significantly attenuated WISP1-induced migration, reducing wound closure by approximately 38% (Figure 7A). These findings indicate that both Src kinases and MIF are involved in the migratory response of non-invasive MCF7 cells induced by WISP1.

To further examine cytoskeletal changes associated with migration, we performed phalloidin staining to visualize filamentous actin. Untreated cells displayed relatively rounded morphology with weaker actin organization (Figure 7B). WISP1 stimulation induced a potent actin remodeling, with elongated cell morphology and enhanced filamentous actin structures at the cell periphery. Co-treatment with PP2 or ISO-1 attenuated these changes, and cells appeared more spread and less polarized compared to WISP1 alone (Figure 7B). Consistently, MIF stimulation induced actin rearrangements and stress fiber formation similar to those observed with WISP1 (Figure 7B).

### 3.8. WISP1 Enhances the Invasive Capacity of MCF7 Cells via Src Kinases and MIF

**Figure 8:**
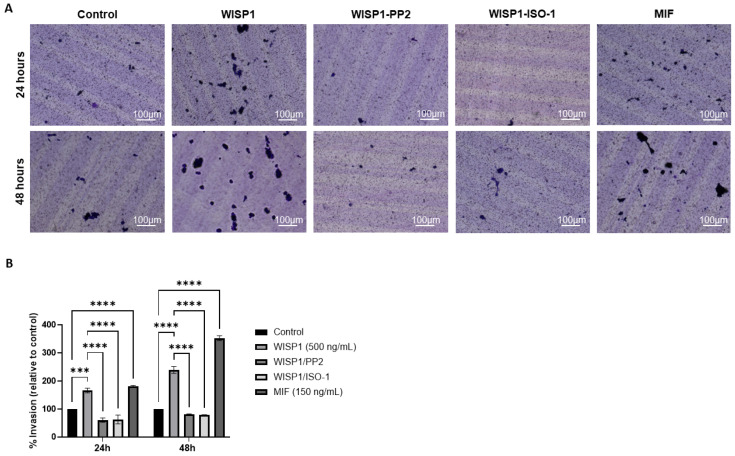
WISP1 promotes the invasiveness of MCF7 cells via Src kinases and MIF. MCF7 cells were treated with WISP1 (500 ng/mL), MIF (150 ng/mL), or pre-treated with the Src kinase inhibitor PP2 (1 μM) or the MIF inhibitor ISO-1 (100 μM) prior to WISP1 stimulation. The 0.1% FBS condition represents the untreated control. (**A**) Invasion was assessed using collagen I-coated Transwell chambers at 24 h and 48 h; invaded cells on the lower membrane surface were fixed, stained with crystal violet, and imaged. (**B**) Quantification of invaded cells, expressed relative to the untreated control, is shown below the representative images. Scale bars, 100 μm. Data are mean ± SD from *n* = 3 independent experiments, each performed in duplicate. Statistical analysis was performed as described in Methods (one-way or two-way ANOVA with Tukey’s multiple comparisons test, as appropriate). *** *p* < 0.001; **** *p* < 0.0001.

To assess whether WISP1-driven molecular changes translate into increased invasive behavior, a collagen-based Transwell invasion assay was performed at 24 h and 48 h. WISP1 significantly increased the invasive capacity of MCF7 cells, with a ~1.7-fold increase at 24 h and a ~2.4-fold increase at 48 h compared to the untreated control (0.1% FBS). Pre-treatment with the Src kinase inhibitor PP2 markedly reduced WISP1-induced invasion by about 64% at 24 h and 62% at 48 h. A similar inhibitory effect of approximately 66% at 24 h and 70% at 48 h was observed with the MIF inhibitor ISO-1, indicating that both Src activity and MIF signaling are required for the pro-invasive actions of WISP1. Consistent with these findings, recombinant MIF alone partially recapitulated the invasive response induced by WISP1, showing a 1.8-fold increase at 24 h and a 3.5-fold increase at 48 h in invasion. Collectively, these results demonstrate that the WISP1/Src/MIF axis promotes the invasive behavior of ER^+^ MCF7 cells (Figure 8).

## 4. Discussion

One of the major hurdles in treating metastatic breast cancer lies in the molecular pathways that promote cancer cell aggressiveness and therapy resistance. This study underscores the critical role of the WISP1/MIF axis in promoting the malignant properties of MCF7 cells, a widely used model for non-invasive estrogen receptor-positive (ER+) breast cancer, and demonstrates how this signaling axis may complicate therapeutic strategies aimed at controlling metastasis and resistance. Given that Src kinases have been implicated in the metastatic process, WISP1 (CCN4) has been increasingly recognized for its oncogenic role across various cancers [19,39], while higher WISP1 expression correlates with advanced disease characteristics, such as larger tumors, lymph node metastasis, and HER-2/neu overexpression [19,40,41,42]. Notably, Src kinases are critical mediators of cell migration and invasion in breast cancer, regulating cytoskeletal reorganization, focal adhesion turnover, and adhesive dynamics that enable metastatic dissemination. The emerging interplay between WISP1 and Src signaling therefore suggests a synergistic mechanism by which these pathways cooperatively enhance breast cancer aggressiveness, particularly by integrating pro-migratory cytoskeletal cues with extracellular matrix remodeling. MCF7 cells express WISP1 that is primarily localized extracellularly, aligning with WISP1’s classification as a secretory matricellular protein [14,35,43]. The presence of WISP1 in non-invasive MCF7 breast cancer cells supports our rationale for utilizing this model.

Our findings show that WISP1 induces the expression of MIF, a cytokine known to be linked to pro-tumorigenic and pro-metastatic activities in various cancers [31,44,45,46,47]. The concentration of 500 ng/mL was identified as optimal for maximal MIF induction in MCF7 cells after 24 h, providing a novel insight into WISP1’s effects in this context. As shown in Figure 1, the induction of MIF by WISP1 is mediated through Lyn and Fyn, but not c-Src, kinase activation. Src kinases facilitate the spread of cancer cells to distant organs and have been implicated in resistance to conventional therapies, such as chemotherapy and hormonal treatments, by sustaining survival signaling pathways in breast cancer cells [48].

Furthermore, our study reveals that MIF itself can regulate its own expression through a feedback loop, since pre-treatment of MCF7 cells with ISO-1, a specific inhibitor of MIF tautomerase activity, resulted in downregulation of the enhanced MIF expression induced by WISP1. These findings indicate that MIF can promote its own expression in response to WISP1 signaling, further reinforcing the role of the WISP1/MIF axis in driving tumor progression and therapy resistance. The above suggests that dual targeting of Lyn/Fyn and MIF may be necessary to fully counteract WISP1-driven metastasis in breast cancer.

Moreover, our findings establish that WISP1 enhances metastatic plasticity in ER^+^ breast cancer by amplifying MIF signaling and engaging Lyn/Fyn-mediated cytoskeletal remodeling, even in low-CD74 contexts. While invasive breast cancers typically rely on high CD74 to maximize MIF-driven EMT (22), our results raise the possibility that ERα^+^ tumors such as MCF7 cells achieve similar outcomes through WISP1-mediated MIF amplification (Figure 1A). Although WISP1 does not directly elevate CD74 (Figure 2C), increased MIF likely strengthens CD74-MIF interactions via mass action in low-CD74 environments [26]. WISP1 also selectively downregulated CD44s without altering total CD44, suggesting a compensatory upregulation of CD44v isoforms. This isoform switching, probably influenced by ERα status [49,50], may couple MIF/CD74 to cofilin-driven actin reorganization and EMT [20,51]. Indeed, WISP1 treatment induced a partial EMT phenotype characterized by reduced membrane E-cadherin, increased fibronectin, and elevated vimentin, consistent with migratory yet adaptable tumor cell states [52,53,54].

Critically, the understanding of how WISP1’s EMT program integrates with Src family kinases, particularly in the context of MIF signaling, highlights the potential for targeted therapies that address both Src kinases and the WISP1/MIF signaling axis. WISP1’s EMT program requires both MIF-CD74/CD44v signaling and Lyn/Fyn activation; MIF maintains mesenchymal traits through pathways previously tied to CD44v-mediated metastasis [12,55,56], while Lyn/Fyn kinases—overexpressed in aggressive breast cancers [33,34,35,36,37,57]—orchestrate E-cadherin internalization and actin remodeling. The functional independence of these arms is evidenced by treatment with either ISO-1 (MIF inhibitor) or PP2 (SFK inhibitor) each reversing EMT markers, suggesting synergistic therapeutic potential. This dual mechanism aligns with established Src-family kinase roles in Fak/Akt/ERK-driven migration [35,40,41] but uniquely positions WISP1 as an upstream integrator of inflammatory (MIF) and cytoskeletal (SFK) pathways in ERα^+^ contexts, suggesting synergistic therapeutic potential. Importantly, the differential inhibitory patterns observed with PP2 and ISO-1 are fully consistent with the hierarchical structure of this signaling axis: PP2 blocks WISP1 signaling at an upstream Lyn/Fyn checkpoint and therefore attenuates both MIF-dependent and MIF-independent outputs, whereas ISO-1 selectively disrupts only the downstream MIF-dependent arm. This supports the idea that WISP1 uses Src-dependent and MIF-dependent signals to regulate different functions in the cell, with Src activity driving receptor expression changes independently of MIF tautomerase activity (Figure 2). Instead, their distinct effects reinforce the notion that WISP1 engages a branched signaling architecture in which Src-linked and MIF-linked pathways operate in parallel as well as cooperatively.

While the interplay between Src and MIF is central to WISP1 signaling, it is also important to acknowledge that WISP1 may exert certain functions through pathways that do not involve either of these two mechanisms. Given the axis’s potential role in promoting EMT, cellular migration, and survival signaling, targeting the WISP1/Src/MIF pathway presents a promising therapeutic approach to combat aggressive ER-Positive breast cancer and may help overcome treatment resistance. Therefore, further exploration of combination therapies that target this signaling axis is warranted and may lead to improved outcomes. Thus, the observed partial or incomplete inhibition does not contradict the existence of a WISP1/Src/MIF axis; rather, it highlights its modular organization and supports a model in which WISP1 drives EMT through multiple, partially overlapping effector routes.

While our data support a model where WISP1-induced CD44 isoform switching synergizes with MIF amplification and SFK activation to drive cytoskeletal reorganization and invasive protrusions in ER^+^ tumors, critical questions persist. Future studies must delineate (1) how ERα regulates CD44 splicing to favor pro-invasive isoforms and (2) whether CD44v-MIF complexes spatially coordinate with Lyn/Fyn kinases at invasion fronts to amplify protrusive activity. Resolving these mechanisms will clarify how WISP1 licenses metastatic plasticity in ERα^+^ contexts and identify strategies to uncouple inflammatory (MIF/CD74) from cytoskeletal (SFK) signaling—a therapeutic opportunity for limiting adaptive aggression in hormonally regulated tumors.

Beyond its role in EMT, WISP1 emerges as a critical regulator of hyaluronan metabolism, linking intracellular signaling to extracellular matrix remodeling in ERα^+^ breast cancer [58,59,60,61]. Our data revealed that WISP1 treatment significantly upregulated the expression of hyaluronan synthase genes *HAS2* and *HAS3*, while suppressed *HYAL2* and induced *HYAL1* and *TMEM2* hyaluronidases. This alteration in hyaluronan metabolizing enzymes suggests that WISP1 not only influences cell signaling pathways but also has profound effects on the tumor microenvironment. These alterations in the hyaluronan metabolizing enzymes led to an increased synthesis of high molecular weight HA (HMW-HA) while allowing the local production of low molecular weight hyaluronan fragments (LMW-HA). Critically, MIF/CD74 signaling sustains this catabolic imbalance, as MIF inhibition restored *HYAL2* expression, whereas Src inhibition attenuated *HAS2/3* induction. This HA landscape creates a hydrated, loose ECM [47,48] that facilitates invasion—a process amplified by CD44v isoforms, which bind HA to activate pro-metastatic pathways [12,47]. Notably, HA fragmentation may further feed forward through CD44v-MIF crosstalk, as LMW-HA enhances CD44 clustering and inflammatory signaling [62,63]. The convergence of WISP1’s transcriptional control over HA metabolism with its regulation of MIF/SFK-driven cytoskeletal remodeling positions it as a central integrator of biochemical and biophysical cues in ER^+^ tumors, while suggesting WISP1-mediated HA remodeling as a potential therapeutic target.

Furthermore, WISP1 appears to act as a regulator of extracellular matrix remodeling in breast cancer, broadly enhancing the expression of matrix metalloproteinases, including MMP1, MMP2, MMP9, and MT1-MMP, and shifting the MMP/TIMP balance toward a proteolytic phenotype [37,38]. This aligns with previous studies reporting that WISP1 promotes MMP2 and MMP9 expression in chondrosarcoma and osteosarcoma cells, thereby facilitating motility and invasion [60]. The upregulation of matrix metalloproteinases (MMPs) highlights WISP1’s role not only in cellular signaling but also in altering the tumor microenvironment to promote invasion. MMP induction is further reinforced by hyaluronan–CD44 interactions, which are known to elevate MT1-MMP levels and contribute to invasive and metastatic phenotypes [64,65,66]. Studies in PC3 prostate cancer cells have shown that WISP1-induced β-catenin signaling has been linked to *HAS2* transcription, suggesting a coordinated regulation of hyaluronan metabolism and MMP activity during ECM remodeling [67]. Src kinase inhibition with PP2 attenuated these effects, indicating that Src kinases are critical mediators of WISP1-induced MMP regulation [33,68,69]. Src is a well-established regulator of MMP expression and activity and often cooperates with FAK to drive cancer cell invasion and migration [70,71]. In particular, Src–FAK signaling upregulates MMP2 and MT1-MMP, which degrade type IV collagen in the basement membrane, a key barrier to cancer invasion [57,72]. Thus, WISP1’s activation of Src facilitates ECM degradation and promotes tumor cell migration, consistent with mechanisms observed in other Src-driven invasive cancer models. Notably, blockade of MIF activity was shown to reduce WISP1-induced MMP1 and MMP2 expression as well as MMP1/TIMP1 and MMP2/TIMP2 ratios, while exogenous MIF enhanced all examined MMPs, suggesting that the WISP1/MIF axis broadly enhances MMP expression, contributing to a pro-invasive phenotype in non-invasive breast cancer cells. However, the further induction of MMP9 and MMP9/TIMP1 ratio upon MIF blockade in the presence of WISP1 implies a rather complex role for MIF in the regulation of MMP9 that needs further investigation.

We also identified NDRG1 as a downstream target repressed by WISP1 and MIF. As a metastasis suppressor that stabilizes E-cadherin and restrains oncogenic pathways (PI3K/Akt, Src, NF-κB) [73,74], its suppression provides a mechanistic link between WISP1 signaling and loss of epithelial integrity. Both Src and MIF inhibition restored NDRG1 expression while attenuating survival, migration, indicating that both pathways act as intermediates in WISP1 signaling. This finding highlights the potential for targeting both the WISP1/Src and WISP1/MIF pathways to enhance NDRG1 function and potentially reduce metastasis. The inverse relationship between NDRG1 expression and cell viability is consistent with reports linking NDRG1 to proliferative and metastatic phenotype [75]. Recombinant MIF alone similarly suppressed NDRG1 and enhanced survival, highlighting its dual role as both a mediator of WISP1 activity and an independent tumor-promoting factor. In parallel, WISP1 was found to significantly enhance breast cancer cell migration, accompanied by cytoskeletal remodeling characterized by actin filament reorganization, stress fiber formation and cell elongation, features typically associated with a motile phenotype. MIF induced a comparable response. These changes were attenuated by Src inhibition or MIF blockade, indicating that the same mechanisms mediating NDRG1 repression also regulate actin organization and motility. Given the central role of cytoskeletal dynamics in metastatic dissemination, these results extend the pro-survival functions of WISP1/Src/MIF axis to induce motility programs that enable invasion. Functionally, WISP1 was shown to enhance the invasive behavior of MCF7 cells, with inhibition of Src kinases or MIF effectively reducing this effect. These findings provide direct evidence that the molecular and cytoskeletal changes induced by WISP1/Src/MIF signaling translate into increased invasive capacity.

Despite providing novel mechanistic insights, this study has some limitations. Experiments were conducted using a single ER^+^ breast cancer cell line (MCF7), and in vivo validation of the WISP1/Src/MIF axis is lacking. Additionally, the effects of this signaling axis in other breast cancer subtypes, such as TNBC, remain unexplored. Future studies should address these gaps by evaluating the relevance of the WISP1/Src/MIF axis across multiple cell lines, patient-derived models, and in vivo systems. Such investigations will clarify how broadly these findings apply and help determine whether targeting WISP1 signaling, alone or in combination with existing therapies, could offer therapeutic benefit across diverse breast cancer contexts.

Moreover, the present data support a translational rationale for therapeutic targeting of the WISP1/Src/MIF axis. Src-family kinases (e.g., Lyn/Fyn), which mediate cytoskeletal remodeling and invasive behavior downstream of WISP1, are already considered druggable targets. Indeed, Src inhibitors have been evaluated in breast cancer. For example, the Src inhibitor Dasatinib showed acceptable tolerability in a Phase II trial in metastatic breast cancer patients [76], while monotherapy with Src inhibitors (e.g., Saracatinib) demonstrated limited efficacy in hormone-receptor negative metastatic disease [77]. Given the dual dependence of WISP1 signaling on both Src-dependent and MIF-dependent pathways, future preclinical and clinical studies should explore combination therapies that simultaneously inhibit Src kinase activity and neutralize the WISP1/MIF axis (e.g., via MIF inhibitors or anti-WISP1 agents). This dual blockade may prove more effective in halting EMT, ECM remodeling, and metastatic progression, especially in therapy-resistant or hormone therapy-refractory tumors.

## 5. Conclusions

This study provides evidence that the WISP1/Src/MIF axis plays a pivotal role in driving the aggressiveness of non-invasive MCF7 breast cancer cells, a widely used cellular model for ER^+^ breast cancer studies, particularly through regulation of cell survival, migration, and ECM remodeling. By activating Lyn/Fyn–MIF signaling, WISP1 amplifies EMT, ECM remodeling, and survival pathways while repressing metastasis suppressors such as NDRG1. MIF cytokine seems to be a major mediator of WISP1-induced changes in cell shape, scattering, proliferation and invasiveness along with the elimination of E-cadherin from the cell membrane. This suggests that specific cues from the tumor microenvironment can activate a migratory/invasive phenotype in a subpopulation of cells residing within the heterogeneous breast tumor. Importantly, the present study highlights the novel contribution of identifying the WISP1/Src/MIF signaling axis as a key regulator of HA metabolism and EMT in ER^+^ breast cancer, providing new mechanistic insights into tumor plasticity and aggressiveness. The present study establishes WISP1 as a nexus linking intracellular signaling to microenvironmental remodeling and identifies Lyn, MIF/CD74, and HA metabolism as promising therapeutic targets in breast cancer. Furthermore, the WISP1/Src/MIF axis may serve as a clinically relevant biomarker to stratify patients and guide targeted therapeutic strategies in ER^+^ breast cancer. Specifically, given the existing clinical development of Src inhibitors and preclinical feasibility of MIF or WISP1 blockade, combination therapies that target both Src kinases and the WISP1/MIF pathway deserve priority. Future studies should evaluate whether targeting WISP1 signaling, alone or in combination with endocrine or chemotherapy, counteracts therapy resistance and improves treatment outcomes in aggressive, hormone therapy-resistant breast cancer.

## Figures and Tables

**Table 1:** Primer sequences used for qPCR.

Gene	Primer Sequence	T_annealing_ (°C)
*CD44s*	F: 5′-ATA ATA AAG GAG CAG CAC TTC AGG A-3′ R: 5′-ATA ATT TGT GTC TTG GTC TCT GGT AGC-3′	60
*CD44t*	F: 5′-ATA ATT GCC GCT TTG CAG GTG TAT T-3′ R: 5′-ATA ATG GCA AGG TGC TAT TGA AAG CCT-3′	60
*CD74*	F: 5′-TGC ATT CAC ATT TGT GCT GTA G-3′ R: 5′-TGT ACA GAG CTC TCC ACG GCT G-3′	60
*E-Cadherin*	F: 5′-TAC GCC TGG GAC TCC ACC TA-3′ R: 5′-CCA GAA ACG GAG GCC TGA T-3′	57
*Fibronectin*	F: 5′-CAT CGA GCG GAT CTG GCC C-3′ R: 5′-GCA GCT GAC TCC GTT GCC CA-3′	57
*GAPDH*	F: 5′-AGG CTG TTG TCA TAC TTC TCA T-3′ R: 5′-GGA GTC CAC TGG CGT CTT-3′	57
*HAS-1*	F: 5′-GGA ATA ACC TCT TGC AGC AGT TTC-3′ R: 5′-GCC GGT CAT CCC CAA AAG-3′	61
*HAS-2*	F: 5′-TCG CAA CAC GTA ACG CAA T-3′ R: 5′-ACT TCT CTT TTT CCA CCC CAT TT-3′	57
*HAS-3*	F: 5′-AAC AAG TAC GAC TCA TGG ATT TCC T-3′ R: 5′-GCC CGC TCC ACG TTG A-3′	61
*Hyal1*	F: 5′-GAT TGC AGT GTC TTC GAT GTG GTA-3′ R: 5′-GGG AGC TAT AGA AAA TTG TCA TGT CA-3′	61
*Hyal2*	F: 5′-CTA ATG AGG GTT TTG TGA ACC AGA ATA T-3′ R: 5′-GCA GAA TCG AAG CGT GGA TAC-3′	61
*MIF*	F: 5′-CCG GAC AGG GTC TAC ATC AAC TAT TAC-3′ R: 5′-TAG GCG AAG GTG GAG TTG TTC C-3′	60
*MMP-1*	F: 5′-TGT GAC CTC CAT CCC CAA CT-3′ R: 5′-AAC TCA GGT CAT CTT CTG TCC GT-3′	57
*MMP-2*	F: 5′-ACT GTT GGT GGG AAC TCA GAA G-3′ R: 5′-CAA GGT CAA TGT CAG GAG AGG-3′	57
*MMP-9*	F: 5′-TTC CAG TAC CGA GAG AAA GCC TAT-3′ R: 5′-GGT CAC GTA GCC CAC TTG GT-3′	57
*MT1-MMP*	F: 5′-ACT GTT GGT GGG AAC TCA GAA G-3′ R: 5′-CAA GGT CAA TGT CAG GAG AGG-3′	57
*TIMP-1*	F: 5′-CGC TGA CAT CCG GTT CGT-3′ R: 5′-TGT GGA AGT ATC CGC AGA CAC T-3′	59
*TIMP-2*	F: 5′-GGG CAC CAG GCC AAG TT-3′ R: 5′-CGC ACA GGA GCC ATC ACT-3′	60
*TMEM2*	F: 5′-GGAATAGGACTGACCTTTGCCAG-3′ R: 5′-TTCTGACCACCCTGAAAGCCGT-3′	57
*Vimentin*	F: 5′-GGC TCG TCA CCT TCG TGA AT-3′ R: 5′-GAG AAA TCC TGC TCT CCT CGC-3′	60

## Data Availability

The data presented in this study are available on reasonable request from the corresponding authors.

## Supplementary Materials

The following supporting information can be downloaded at: https://www.mdpi.com/article/10.3390/cells15020160/s1, Table S1. Reagents used in this study. Figure S1: CD74 mRNA expression following WISP1 and Src/MIF inhibitors treatment. Figure S2: Effects of WISP1 and MIF on MMP/TIMP mRNA levels. Figure S3: Quantification of wound closure assessed by wound-healing assay at 0, 6, 12, 24, and 48 h. Figure S4: Representative phase-contrast images of the wound-healing assays acquired at 0, 6, 12, 24, and 48 h.
