# Supplementary material for: The WISP1/Src/MIF Axis Promotes the Malignant Phenotype of Non-Invasive MCF7 Breast Cancer Cells
Source: Cells. 2026 Jan 15;15(2):160. doi: 10.3390/cells15020160 (PMC12839993; doi:10.3390/cells15020160)
Supplement: Supplementary file 1 [file cells-15-00160-s001.zip › Supplementary Figures.docx]

**
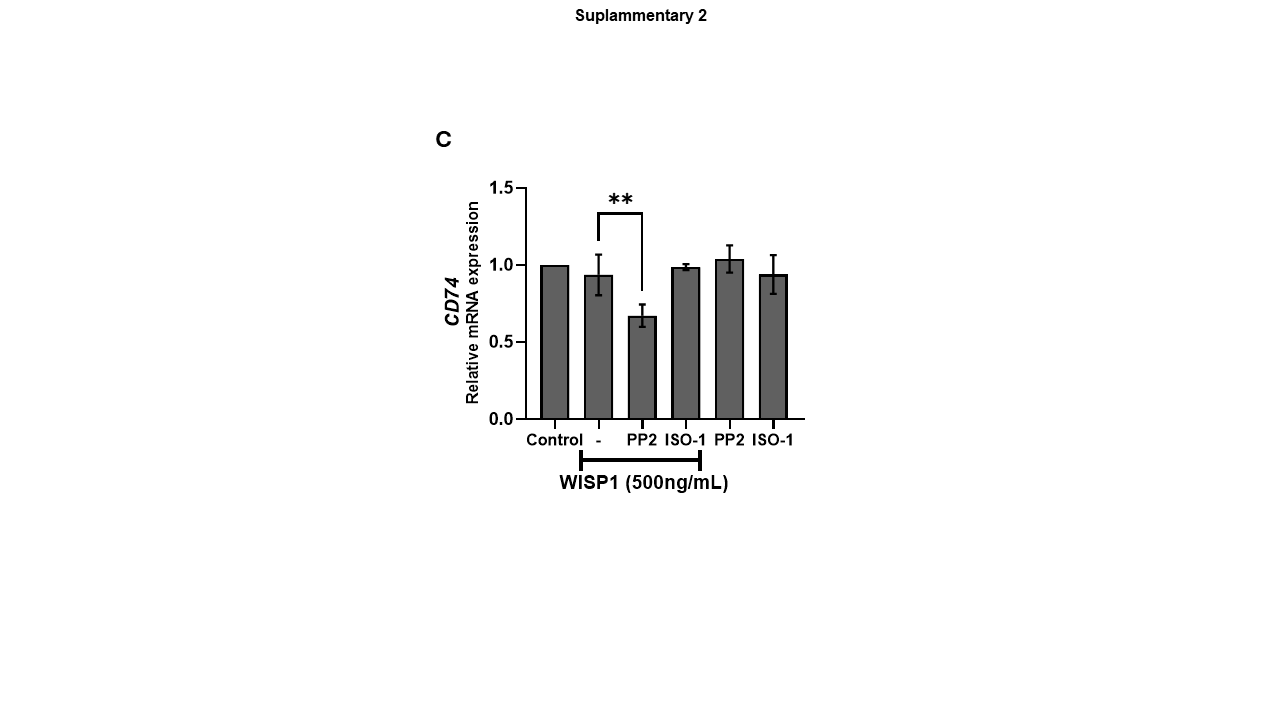
**

**Supplementary Figure S1 | CD74 mRNA expression following WISP1 and Src/MIF inhibitors treatment.**
MCF7 cells were treated for 24 h with WISP1 (500 ng/mL), PP2 (1 µM), or ISO-1 (100 µM), alone or in combination as indicated. CD74 mRNA levels were quantified by qPCR, normalized to GAPDH, and expressed relative to untreated control. Data are mean ± SD from n = 3 independent experiments, each performed in duplicate. Statistical analysis was performed as described in Methods. **P < 0.01.

**
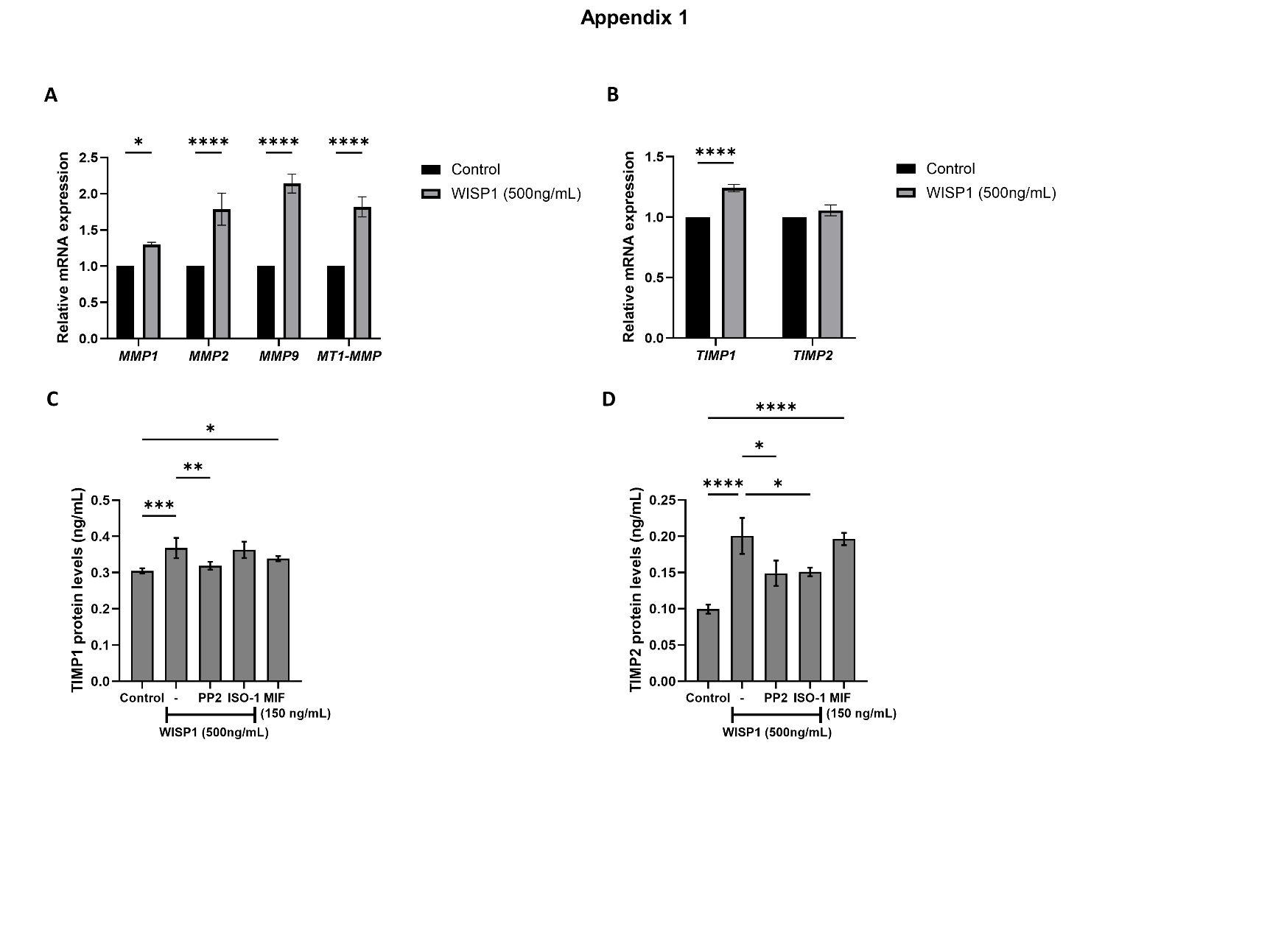
**

**Supplementary Figure S2 | Effects of WISP1 and MIF on MMP/TIMP mRNA levels**. MCF7 cells were treated for 24 h with WISP1 (500 ng/mL). Where indicated, cells were co-treated with the Src family kinase inhibitor PP2 (1 µM) or the MIF inhibitor ISO-1 (100 µM); a recombinant human MIF (rhMIF, 150 ng/mL) condition was included as a comparator. Gene expression analysis of (A) MMP1, MMP2, MMP9, MT1-MMP; (B) TIMP1, TIMP2 by qPCR, normalized to GAPDH and expressed relative to untreated control. ELISA quantification of TIMP1 (C), TIMP2 (D) protein levels (ng/mL). **Data are mean ± SD from n = 3 independent experiments, each performed in duplicate.** Statistical analysis was performed as described in Methods (one-way or two-way ANOVA with Tukey’s multiple comparisons test, as appropriate). *P < 0.05; **P < 0.01; ***P < 0.001; ****P < 0.0001.

**
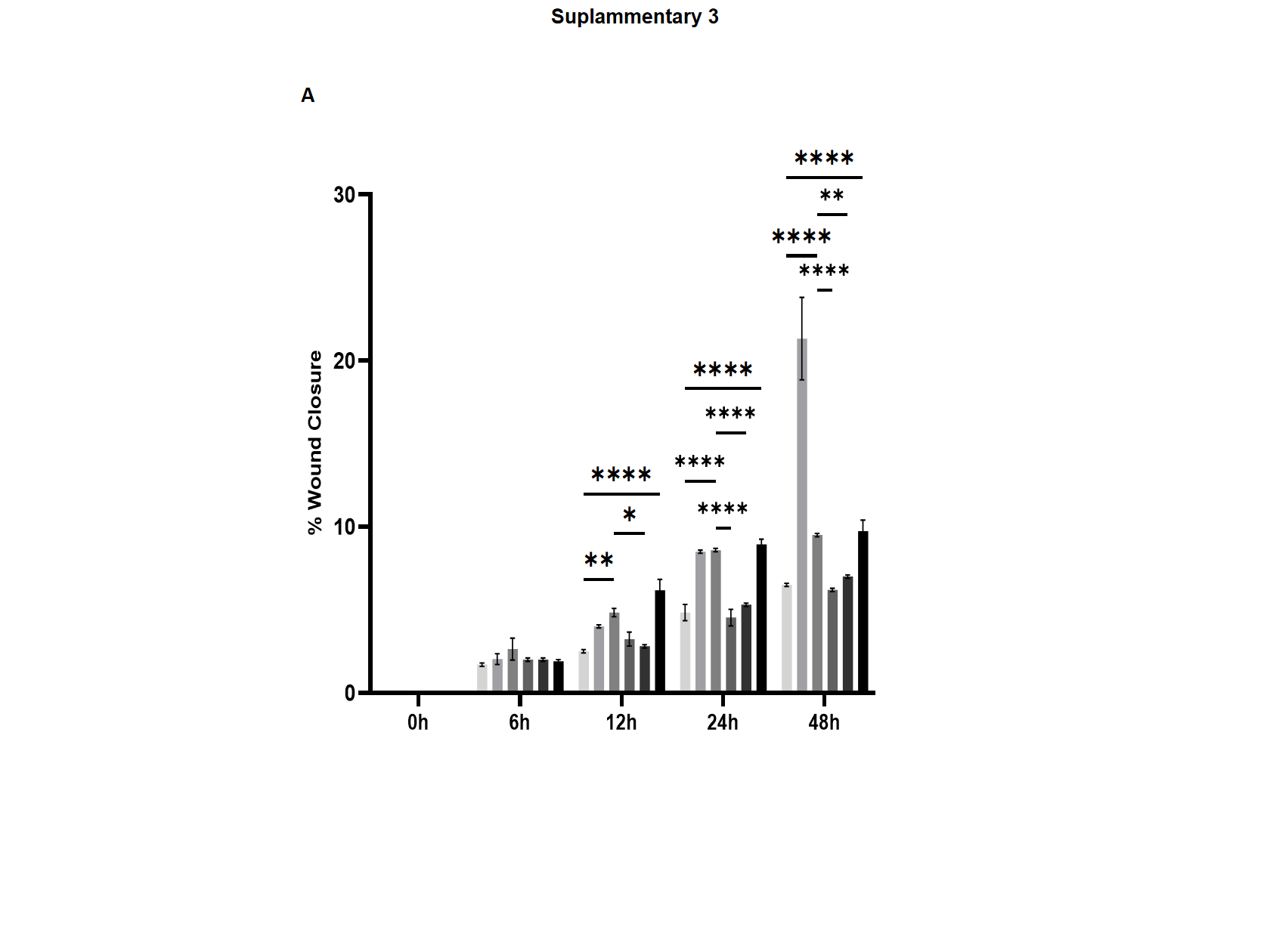
**

**Supplementary** **Figure S3 | Quantification of wound closure assessed by wound-healing assay at 0, 6, 12, 24, and 48 h.** MCF7 cells were treated with WISP1 (500 ng/mL) or MIF (150 ng/mL), or pre-treated with the Src kinase inhibitor PP2 (1 µM) or the MIF inhibitor ISO-1 (100 µM) prior to WISP1 stimulation. Wound closure is expressed as the percentage of the initial wound area at 0 h. Data are mean ± SD from n = 3 independent experiments, each performed in duplicate. Statistical analysis was performed as described in Methods (one-way or two-way ANOVA with Tukey’s multiple comparisons test, as appropriate). *P < 0.05; **P < 0.01; ****P < 0.0001.

**
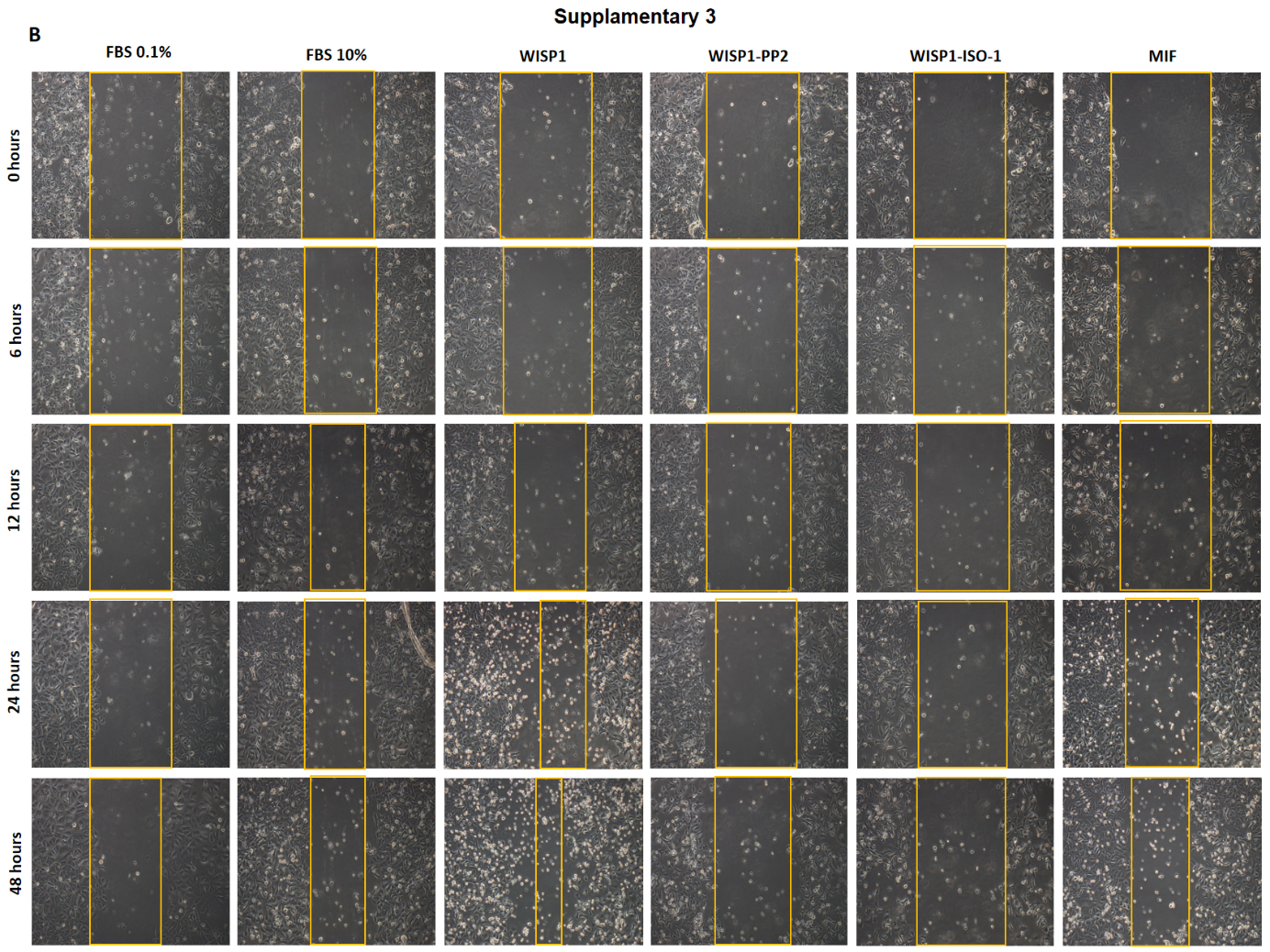
**

**Supplementary** **Figure S4 |** Representative phase-contrast images of the wound-healing assays acquired at 0, 6, 12, 24, and 48 h. MCF7 cells were treated with WISP1 (500 ng/mL) or MIF (150 ng/mL), or pre-treated with the Src kinase inhibitor PP2 (1 µM) or the MIF inhibitor ISO-1 (100 µM) prior to WISP1 stimulation.
