# Supplementary material for: The WISP1/Src/MIF Axis Promotes the Malignant Phenotype of Non-Invasive MCF7 Breast Cancer Cells
Source: Cells. 2026 Jan 15;15(2):160. doi: 10.3390/cells15020160 (PMC12839993; doi:10.3390/cells15020160)
Supplement: Supplementary file 1 [file cells-15-00160-s001.zip › Supplementary Table S1.docx]

**Supplementary** **Table S1. Reagents used in this study**

| **Reagent / Kit** | **Supplier** | **Catalog Number** |
| --- | --- | --- |
| 4,5-Dimethylthiazol-2-yl)-2,5-diphenyltetrazolium bromide (MTT) | Sigma‑Aldrich, Merck KGaA, Darmstadt, Germany | 475989 |
| Alexa Fluor 488-conjugated secondary antibody | Thermo Fisher Scientific GmbH, Dreieich, Germany | A-11008 |
| α-Tubulin antibody | Invitrogen, Thermo Fisher Scientific GmbH, Dreieich, Germany | 322500 |
| anti-mouse IgG | Sigma‑Aldrich, Merck KGaA, Darmstadt, Germany | A4416 |
| anti-rabbit IgG | Sigma‑Aldrich, Merck KGaA, Darmstadt, Germany | A0545 |
| Anti-phosphotyrosine HRP | Cytoskeleton, Inc., Denver, CO, USA | APY03-HRP |
| Anti-c-Src antibody | Proteintech Group, Inc., Rosemont, IL, USA | 66606-1-Ig |
| Anti-Fyn antibody | Proteintech Group, Inc., Rosemont, IL, USA | 11097-1-AP |
| Anti-Lyn antibody | Proteintech Group, Inc., Rosemont, IL, USA | 20415-1-AP |
| Bradford Protein Assay Kit | Thermo Fisher Scientific GmbH, Dreieich, Germany | 23236 |
| BCA Protein Assay Kit, Pierce | Thermo Fisher Scientific GmbH, Dreieich, Germany |  |
| Collagen I, High Concentration, Rat Tail, 100 mg | **Corning, Corning GmbH, Kaiserslautern, Germany** | 354249 |
| Cytarabine | Sigma‑Aldrich, Merck KGaA, Darmstadt, Germany | C9768 |
| DMEM | Biosera, Cholet, France | LM-D1110/500 |
| E-Cadherin (24E10) Rabbit Monoclonal Antibody | Cell Signaling Technology, Danvers, MA, USA | 3195 |
| Human MMP-1 ELISA Kit | Bio-Techne GmbH, Wiesbaden‑Nordenstadt, Germany | EHMMP1 |
| Human MMP-2 (total) ELISA Kit | Bio-Techne GmbH, Wiesbaden‑Nordenstadt, Germany | MMP200 |
| Human MMP-9 ELISA Kit | Thermo Fisher Scientific GmbH, Dreieich, Germany | BMS2016-2 |
| Human MT1-MMP ELISA Kit | Bio-Techne GmbH, Wiesbaden‑Nordenstadt, Germany | EEL068 |
| Human TIMP-1 ELISA Kit | Bio-Techne GmbH, Wiesbaden‑Nordenstadt, Germany | DTM100 |
| Human TIMP-2 ELISA Kit | Bio-Techne GmbH, Wiesbaden‑Nordenstadt, Germany | DTM200 |
| Human MIF ELISA Kit | Bio-Techne GmbH, Wiesbaden‑Nordenstadt, Germany | DY289 |
| Immunobilon® Crescendo Western HRP Substrate | Merck Millipore, Darmstadt, Germany | WBLUR0100 |
| Fetal Bovine Serum (FBS) | Biosera, Cholet, France | FB-1000/500 |
| Hyaluronan ELISA Kit | Bio-Techne GmbH, Wiesbaden‑Nordenstadt, Germany | DHYAL0 |
| HRP-conjugated streptavidin | R&D Systems, Wiesbaden‑Nordenstadt, Germany | DY998 |
| HRP-conjugated anti-mouse IgG | Sigma‑Aldrich, Merck KGaA, Darmstadt, Germany | A4416 |
| HRP-conjugated anti-rabbit IgG | Sigma‑Aldrich, Merck KGaA, Darmstadt, Germany | A0545 |
| ISO-1 (MIF inhibitor) | Tocris Bioscience, Bristol, UK | **4288** |
| KAPA SYBR FAST qPCR Master Mix (2×) kit | Kapa Biosystems, Woburn, MA, USA | KK4602 |
| MIF antibody | Proteintech Group, Inc., Rosemont, IL, USA | 20415-1-AP |
| NDRG1 antibody (HRP-conjugated) | Novus Biologicals, Centennial, CO, USA | NB160805 |
| Nucleo Spin RNA kit | Macherey‑Nagel, Düren, Germany | **ML‑8761** |
| PBS | Biosera, Cholet, France | LM-T1706/500 |
| Phalloidin-iFluor 488 | AAT Bioquest, Sunnyvale, CA, USA | 23115 |
| protease and phosphatase inhibitor cocktail | Merck Millipore, Darmstadt, Germany | 20-201 |
| PP2 (Src kinase inhibitor) | Tocris Bioscience, Bristol, UK | 1407 |
| Prime Script RT Reagent Kit | Takara, Kusatsu, Shiga, Japan | RR047A |
| Recombinant human WISP1 | PeproTech, Rocky Hill, NJ, USA | **120‑18** |
| Recombinant human MIF | ImmunoTools, Friesoythe, Germany | **11344263** |
| RIPA buffer | Chemicon/Millipore, Burlington, MA, USA | 20-201 |
| PVDF membranes | MACHEREY‑NAGEL, Düren, Germany | 729219 |
| Trypsin-EDTA 1× | Biosera, Cholet, France | LM-T1706/500 |
| Src antibody (mouse monoclonal) | Proteintech Group, Inc., Rosemont, IL, USA | 66606-1-Ig |
| Vectashield Antifade Mounting Medium with DAPI | Vector Laboratories, Burlingame, CA, USA | H-1200 |
